# Modern Sociology

**Published:** 1903-01-03

**Authors:** 


### MODERN SOCIOLOGY.

### THE CANTEEN IN THE NAVY.

"LIFE on board a battleship with 700 or 800 men under the iron system of necessary discipline is hard to bear at the best of times. On board a small ship or a destroyer it is intolerable in bad weather and detestable always. The pleasures of existence are few. Perhaps the chief of them is the hope that comes from the knowledge of good work well done. The second is food." Thus says Mr. Arnold White in a recent number of the "National Review," and he goes on to show that this second alleviation of the lot of the tar is but sparingly administered.

The men on board a man-of-war get three meals a day, the last of which is served at a quarter past four in the afternoon. His country allows Jack nothing more until breakfast time the following morning. If he wants something to eat before turning in he can get it, to be sure, but he must buy it at the canteen on board the ship and pay for it out of his own pocket. This seems hard. We should none of us like to be compelled to fast from afternoon tea-time until breakfast time next morning—even those of us who are not doing active work in the bracing atmosphere of the sea, which admittedly tends to encourage appetite. And we should all resent it if, having accepted a situation with "all found," as the maid-servants put it, we found that the food provided was not sufficient to maintain us in full physical efficiency, and without feeling the pangs of hunger in an acute form. The canteen is meant to provide little luxuries, which are with reason not included in the Government rations, for those who care to spend money on them; but it is not fair that the food provided should be so scanty that the seaman should be compelled by absolute hunger to eke out his rations by personal purchases. Yet this is what is almost universally done. The men—seamen, mechanics, stokers—spend from sixpence to eightpence a day out of their wages on additions to the food provided for them. This three to four shillings a week must be deducted from the amount payable to the wife on shore, and if Jack is well fed, Jill must stint.

Moreover, there is no fixed canteen price. The charge made for different articles varies on board different ships. Cheese, of which the average price was 8d. per lb. on a number of ships, rose to 50 cents.—2s.—on board the *Terrible*. On the same ship flour—and why should a sailor have to buy flour?—costs 40 cents., or 1s. 8d. a gallon, while on the *Revenge* the price was 9d. a gallon. Currants vary from 3½d. to 6d. a lb., but one would not pay too much heed to the question of currants, or raisins, or pickles, or sardines, which are more or less of the nature of luxuries. Yet in common fairness, men doing the same work and receiving equal wages, should not have to pay different prices for the same commodities. Should there not be a Government price for the things, which should charge no profit beyond what is necessary to pay the expense of the canteen? Why, if Jack does want more than is provided for him, should he be exploited by the keeper of the canteen? Since from the nature of his case, he has no choice of shops, it becomes the duty of his employers to see that the shop, where he is compelled to deal, is fairly conducted. It would be unjust to say that the canteen is always ill-managed. Of one a man who deals with it says, "There is no fault to find with the canteen. The articles sold are of the very best The way it is conducted reflects credit on all connected with it. It is a God-send to us in the Persian Gulf, where the meat is of the poorest quality and vegetables unheard of." But concerning another canteen this is the testimony: "Flour is very bad. Bread one day old is sour. Sugar nearly always wet, and though sold in canteen comes from the ship's stores. Baking and egg powder perished. Cannot be sold anywhere else, but does very well for sailors. The socks are only good to look at. Everything that is sold by the pound is 12 ounces to the pound." Thus it seems to be pretty much a matter of luck whether a man has to pay a reasonable sum for good articles or an exorbitant one for bad ones. It is impossible in a service which girdles the globe to make the conditions of service exactly equal for all, but in this matter of the canteen there is surely a possibility of improvement. These canteens are sometimes "run" by private firms and sometimes by a committee of the men on board, supervised by one of the officers. Neither arrangement is invariably satisfactory. The popular control which the committee is supposed to supply is said to be occasionally little more than a farce, since complaints may be twisted into evidences of disrespect, which is punished as a crime. Indeed, cases have been known where petty officers have been warned that it will be well for them not to grumble, if they wish to keep out of trouble. To make it more difficult for petty officers to be scrupulously fair in judging of the management of the canteens, they are sometimes bribed by those who are running them. One who is quoted by Mr. White admits this. "I must honestly say," he writes, "that if the canteens were fairer to broadside messes the P.O.'s would not get 1s. in the £ discount, while the others only get 6d. I know this for a fact, as I have been caterer in first class P.O's mess for eighteen months, and we used to get fruit gratis, but that was too open, so they stopped that for a 10 lb. ham every month; also cleaning gear, paste, emery, brick, soda, soap, besides numerous other things. These were, of course, a bribe to keep the P.O.'s from complaining." We might wonder how emery powder, soap, and soda could be a bribe to any man, if we did not know that the economy of the authorities makes it necessary for all officers, from the captain down, to pay for much in the way of painting and cleaning out of their own pockets, if they want their ship to look as smart as we expect everything in the Navy to be. It is part of the system which, in both services, makes it difficult for an officer to live on his pay—a system which must always keep out many competent men.

The results of the investigation of the Committee on Navy Rations promises to mend matters in some degree. The present three meals a day are to be—at some date next year which is not yet fixed—increased to five, and another hour is to be allowed for eating them. Thus, it is to be hoped, the necessity for the men to go to the canteen for victuals to eke out their insufficient rations will be done away with. But the canteen itself is to remain. Nor were the Committee permitted to advise the placing of the canteens directly under Government. One can see how much inconvenience might be caused by the necessity of referring every point in the management of a canteen in, say, the China Sea, to an authority in London; but on the other hand the leaving of the canteen under the control of a private adventurer must perpetuate many of the evils at present complained of. But the increase in the rations provided is a thing to be thankful for. Recruiting for the Navy does not go on as well as one would desire, considering the importance of an efficient Navy to the Empire. With the ever-growing need for skilled men as mechanics and gunners on board our ships, it is important that recruits should be drawn from a good class. The class of men wanted would get good wages on shore, and would eat plenty of good food. If they cannot get that in the Navy will they join it?

